# ESPO AND NIA’S BUTLER WILLIAMS PROGRAM: RESOURCES AND SUPPORTS TO CULTIVATE THE FORTITUDE FACTOR

**DOI:** 10.1093/geroni/igae098.1021

**Published:** 2024-12-31

**Authors:** Jaime Hughes, Rita Hu, Patricia Jones

**Affiliations:** Chair; Co-Chair; Discussant

## Abstract

Aging research, education, and practice all continue to expand in their diversity across methods, scope, and reach. Navigating a career in any of one of these sectors requires many elements of fortitude – curiosity, discipline, collaboration, creativity, and resilience. The Gerontological Society of America’s Emerging Scholar and Professional Organization (ESPO) has long collaborated with the National Institute on Aging to support students, trainees, and emerging professionals in their scientific and professional development. The Butler Williams Scholars Program, hosted by the NIA’s Office of Special Populations, is an annual immersive program for advanced postdoctoral fellows and early career faculty. For many attendees, participating in this program is an opportunity to engage with Program Officers, exchange ideas among colleagues with diverse training and interests, and develop a national network of scientific and professional collaborators. This symposium features work by past Butler Williams participants, including reflections on how GSA and NIA have each helped to shape professional and research trajectories. Specifically, Dr. Matos-Moreno will discuss ongoing work around kinship structures in different migratory scenarios. Dr. Hughes will discuss a portfolio of interdisciplinary research focused on the scale and spread of health promotion programs for older adults. Dr. Dotson will discuss a career composed of diverse funding mechanisms and initiatives to promote healthy lifestyles and optimize brain health. The session will close with remarks by staff from NIA’s Office of Special Populations and other program areas.

